# Simultaneous gene silencing of Bcl-2, XIAP and Survivin re-sensitizes pancreatic cancer cells towards apoptosis

**DOI:** 10.1186/1471-2407-10-379

**Published:** 2010-07-20

**Authors:** Felix Rückert, Nicole Samm, Anne-Kathrin Lehner, Hans-Detlev Saeger, Robert Grützmann, Christian Pilarsky

**Affiliations:** 1 Department of Visceral, Thoracic and Vascular Surgery, University Hospital Carl Gustav Carus, Technische Universität Dresden, Fetscherstrasse 47, 01307 Dresden, Germany

## Abstract

**Background:**

Pancreatic ductal adenocarcinoma shows a distinct apoptosis resistance, which contributes significantly to the aggressive nature of this tumor and constrains the effectiveness of new therapeutic strategies. Apoptosis resistance is determined by the net balance of the cells pro-and anti-apoptotic "control mechanisms". Numerous dysregulated anti-apoptotic genes have been identified in pancreatic cancer and seem to contribute to the high anti-apoptotic buffering capacity. We aimed to compare the benefit of simultaneous gene silencing (SGS) of several candidate genes with conventional gene silencing of single genes.

**Methods:**

From literature search we identified the anti-apoptotic genes XIAP, Survivin and Bcl-2 as commonly upregulated in pancreatic cancer. We performed SGS and silencing of single candidate genes using siRNA molecules in two pancreatic cancer cell lines. Effectiveness of SGS was assessed by qRT-PCR and western blotting. Apoptosis induction was measured by flow cytometry and caspase activation.

**Results:**

Simultaneous gene silencing reduced expression of the three target genes effectively. Compared to silencing of a single target or control, SGS of these genes resulted in a significant higher induction of apoptosis in pancreatic cancer cells.

**Conclusions:**

In the present study we performed a subliminal silencing of different anti-apoptotic target genes simultaneously. Compared to silencing of single target genes, SGS had a significant higher impact on apoptosis induction in pancreatic cancer cells. Thereby, we give further evidence for the concept of an anti-apoptotic buffering capacity of pancreatic cancer cells.

## Background

An important feature of oncogenesis is the alteration of cell signaling pathways, resulting in certain cancer phenotypes [1]. Pancreatic ductal adenocarcinoma (PDAC) is an extremely aggressive tumor that ranks eighth among the most common cancers in the Western world [2].

Recently, comprehensive analysis of the genome and the transcriptome of pancreatic tumors demonstrated the importance of the apoptosis pathway in the pathophysiology of pancreatic cancers [3,4]. Clinicopathologically, apoptosis resistance contributes to the tumor's poor response to chemotherapy, ionizing radiation and immunotherapy [5] and affects the metastasizing capacity and growth rate of the tumor [6,7]. Apoptosis resistance therefore seems to be the hallmark of cancer that largely accounts for the aggressive nature of pancreatic cancer.

Two distinct pathways regulate Apoptosis. The 'intrinsic' pathway is the primary death program responsive to different cell stress stimuli, with the mitochondrion as the central conduit. The 'extrinsic' cell death pathway is activated through death receptors. Many physiological control points protect the cell from inappropriate apoptosis induction [8]. The net balance of all pro-and anti-apoptotic "control mechanisms" determines the apoptotic threshold of the cell. Apoptosis occurs when the pro-apoptotic load of the cell exceeds its anti-apoptotic buffering capacity [9].

Because of its clinical importance, the re-engagement of the disrupted apoptosis program in pancreatic cancer offers a compelling general strategy for effective and tumor-cell-specific cancer therapy [3]. Previous studies targeting single apoptosis-associated genes in pancreatic cancer have shown encouraging results [10,11].

However, targeting different apoptosis-associated genes simultaneously seems even more promising.

First, pancreatic cancer displays a large number of dysregulations within the cell death signaling pathway, resulting in a high anti-apoptotic buffering capacity [8,12]. Secondly, the pathway is generally characterized by redundancy that compensates for perturbations affecting individual components [9].

The aim of this study was to identify important dysregulated signaling interfaces of the apoptosis pathway and to test whether simultaneous gene silencing (SGS) of those candidate genes had a synergistic effect on the apoptotic threshold of tumor cells *in vitro*. Bcl-2, XIAP and Survivin were identified as overexpressed, synergistic and anti-apoptotic members of the pathway by literature search [11,13]. We performed SGS in two cell lines, MiaPaCa-2 and AsPC-1, which displayed slightly different expression of the target genes. Effectiveness of SGS was assessed by qRT-PCR and western blotting. SGS reduced the apoptotic threshold of both cell lines significantly as measured by flow cytometry and caspase activation. Based on our results we conclude that SGS is an effective method for re-sensitizing pancreatic cancer cells towards apoptosis.

## Methods

### Identification of apoptosis-associated genes

Keywords for the search were "apoptosis", "cell death", "cell death pathway", "cell death receptor" together with the term "pancreatic carcinoma" or "pancreatic cancer". Genes were considered if dysregulation was demonstrated on the level of the transcriptome and the proteome. The literature search comprised publications until October 2008.

### Cell culture, transfection conditions and simultaneous gene silencing assay

The MiaPaCa-2 cell line (ATCC: CRL-1420), derived from a primary pancreatic carcinoma, was used for this study. Cells were grown in DMEM with high glucose, 1.5 g/l sodium bicarbonate and 4 mM GlutaMAX (Invitrogen, Karlsruhe, Germany) with 10% FCS and 2.5% horse serum. We also used the AsPC-1 cell line (ATCC: CRL-1682), derived from malignant ascites. Cells were grown in RPMI-1640 medium with 2 mM GlutaMAX, 1 mM sodium pyruvate, 4.5 g/l glucose (Invitrogen, Karlsruhe, Germany) and 10% FCS. Both cell lines were cultured in a humidified atmosphere containing 5% CO_2 _at 37°C. Next, 5 × 10^4 ^cells were transfected using Oligofectamine (Invitrogen GmbH, Karlsruhe, Germany). Target sense sequences that effectively mediated silencing were as followed: *Bcl-2 *[14]: GUACAUCCAUUAUAAGCUG (NCBI probe ID: Pr196034.1), *XIAP *[15]: GUGGUAGUCCUGUUUCAGC, *Survivin*: GAAUUUGAGGAAACUGCGA [16] (all from MWG Biotech, Ebersberg, Germany). As negative control, we used siRNA against Firefly Luciferase (CGUACGCGGAAUACUUCGA), the Allstars siRNA (Qiagen) and carrier solution. A total of 50,000 cells/well were single-transfected with 0.046 nM of two negative controls (nonsense siRNA); one group was treated with vehicle only. For the SGS, cells were co-transfected with 0.015 nM of each of the three target genes. Additional controls were performed using single transfection of the different target genes, either with low-dose siRNA (0.015 nM) or standard-dose siRNA (0.046 nM). The low-dose single transfection (lowST) was undertaken with 0.015 nM Bcl-2, Survivin or XIAP siRNA, each with 0.03 nM of nonsense siRNA to exclude bias caused by the absolute amount of siRNA. Standard-dose single transfection (ST) was undertaken with 0.046 nM of the correspondent target gene. Cells were cultured for 72 h. At the end of incubation, all cells were harvested. After treatment of the cells, flow cytometry and a caspase activity assay were performed to determine the apoptosis rate. Silencing was confirmed by western blot.

### Reverse Transcription Polymerase Reaction (RT-PCR)

For quantitative RT-PCR, total RNA was isolated using the RNeasy Mini Kit (Qiagen, Hilden, Germany), cDNA was synthesized using random primer and SuperScript II (Invitrogen GmbH, Karlsruhe, Germany). The genes were amplified with the Power SybrGreen PCR Master Mix according to the manufacturer's instructions. Gene expression was quantified by the comparative cT-Method, normalizing cT-values to a housekeeping gene (β-Actin) and calculating the relative expression values. Primers used in this study are shown in table 1.

**Table 1:** Primers used for RT-PCR in the present study

Primer	Forward	reverse
β-Actin	AAGCCACCCCACTTCTCTCTAA	AATGCTATCACCTCCCCTGTGT
Bcl-2	ATGTGTGTGGAGAGCGTCAA	ACAGTTCCACAAAGGCATCC
Survivin	GTTGCGCTTTCCTTTCTGTC	TCTCCGCAGTTTCCTCAAAT
XIAP	CACTTGAGGTTCTGGTTGCAG	TGCAAAGCTTCTCCTCTTGC
STAT1	AATGCTGGCACCAGAACGAA	ATCACCACAACGGGCAGAGA
IFNB	GCAATTGAATGGGAGGCTTG	GGCGTCCTCCTTCTGGAACT

### Western Blotting

Cells were lysed with LDS sample buffer (Invitrogen, Karlsruhe, Germany). Proteins were electrophoresed under reducing conditions on 4-12% acrylamide gels (Invitrogen, Karlsruhe, Germany) and then transferred to a nitrocellulose membrane (Hybond ECL, GE Healthcare, Munich, Germany). To block nonspecific binding, the membrane was incubated overnight in TBS-T 0.1% containing 5% BSA for Bcl-2 or 5% milk for Survivin and XIAP at 4°C. Subsequently, the membrane was either incubated with an antibody to *Survivin *(1:1000, NB 500-201, Novus Biologicals, Acris Antibodies, Hiddenhausen, Germany), *Bcl-2 *(1:200, sc-509, Santa Cruz, Heidelberg, Germany), *XIAP *(1:500, 610716, Becton Dickinson, Heidelberg, Germany) or *GAPDH *(1:5000, ab 8245-100, abcam, Cambridge, UK) in TBS-T 0.1% with blocking as mentioned above for 1 h. After washing in TBS, the protein was visualized using the ECL detection kit (GE Healthcare, Munich, Germany) with a peroxidase-labeled anti-mouse antibody (1:25,000, NIF825, GE Healthcare, Munich, Germany) or an anti-rabbit antibody (1:5,000, NIF824, GE Healthcare, Munich, Germany).

For a loading control, the membranes were blocked again and probed with monoclonal antibodies for *GAPDH *according to standard protocols. Protein expression was determined as the ratio of target gene staining intensity to *GAPDH *staining intensity using AIDA evaluation software (Raytest, Straubenhardt, Germany). To compare blots of the different transfections, staining intensities of the different transfections were normalized to the SGS treated group. All blots were done in triplicate.

### Determination of cell death and apoptosis

Cells were harvested 72 h after transfection. Cells were trypsinized and treated with 50 μg/mL propidium iodide and Annexin V-FITC (Becton Dickinson, Heidelberg, Germany). The cells were analyzed and quantified with flow cytometry (10,000 cells analyzed) (FACS Calibur; Becton Dickinson, Heidelberg, Germany). For analysis of Apoptosis we counted Annexin-positive cells of the upper and lower right quadrants. For further statistical analysis, we formed a quotient with untreated cells for every group. Caspase 3 and 7 activity was measured on deep frozen cell pellets containing 20,000 cells harvested 72 h after transfection using the Caspase Glo-Assay (Promega, Mannheim, Germany) according to the manufacturer's instructions. Results of the caspase assay were also normalized to untreated cells.

### Statistical analysis

For statistical analysis, the t test and the Mann-Whitney U test of "SPSS 13.0" for Windows were used.

## Results

### Selection of target genes

A comprehensive literature search identified several dysregulated apoptosis-associated genes in pancreatic cancer. The ideal characteristics of our candidate genes for SGS were upregulation at the RNA and protein levels and synergistic anti-apoptotic action in the course of the cell death pathway. Because the intrinsic pathway has a central role in pancreatic cancer cells we only considered members of this pathway for our study. XIAP [13,17,18], Survivin [11,13,19] and Bcl-2 [20-22] met all these criteria and are characterized as upregulated in PDAC cell lines as well as native tumor tissue.

### Simultaneous gene-silencing of Bcl-2, Survivin and XIAP

**Figure 1:**
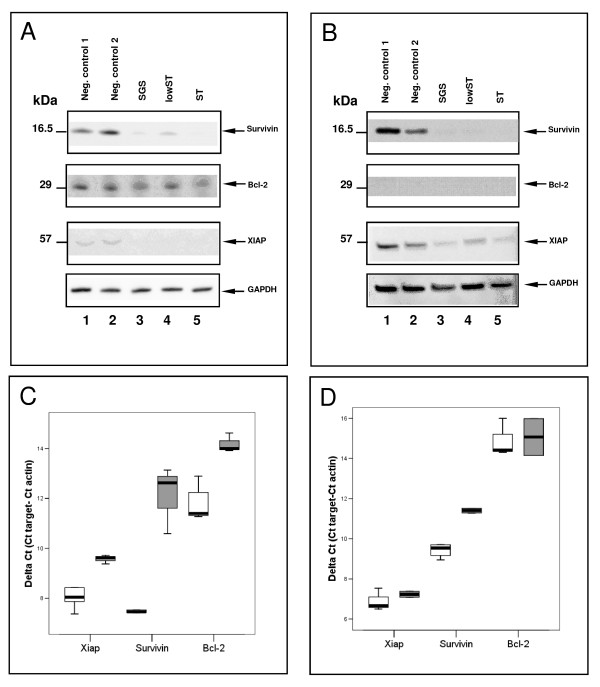
**Gene-silencing of Bcl-2, Survivin and XIAP**. Western blot showed efficient silencing in transfected MiaPaCa-2 (**A**) and AsPC-1 cells (**B**). 50,000 cells/well were single-transfected with carrier solution (lane 1) and siRNA against Luciferase (lane 2) as control. SGS, lowST and ST all effectively silenced the three target genes (lane 3-5). Efficient knock-down was also shown in the lowST group by RT-PCR in MiaPaCa-2 (**C**) and AsPC-1 cells (**D**). White bars show controls, grey bars signify transfected cells. All samples were normalized to β-Actin as a house-keeping gene. SGS = Simultaneous gene silencing; lowST = Low dose siRNA transfection; ST = Standard dose siRNA transfection.

To elucidate the effect of simultaneous gene-silencing in pancreatic cancer, we established a protocol for simultaneous post transcriptional gene silencing using siRNA. For SGS we chose the human pancreatic cancer cell line MiaPaCa-2, which expresses XIAP, Bcl-2 and Survivin and AsPC-1 cells, which express XIAP and Survivin (Figure 1; Additional file 1). We determined the maximal dose without toxic effects to be 0.046 nM siRNA per 50,000 cells (data not shown). Using qRT-PCR and western blot we verified the target genes to be silenced at the mRNA and protein levels in transfected cells. QRT-PCR showed the expression of all three target genes in SGS, lowST and ST to be strongly reduced compared with controls. We validated knock-down of the target genes by SGS in western blot.

The reduction of Bcl-2, XIAP and Survivin protein expression by SGS was statistically significant as compared to controls in MiaPaCa-2 cells (p < 0.05). We also found a statistically significant reduction of the expression of XIAP and Survivin by SGS in AsPC-1 cells in western blot (p < 0.05) (Figure 1).

LowST and ST transfection also resulted in a significant reduction of protein expression of the target genes in both cell lines in western blot.

To examine off-target effects of gene-silencing in our cells, we performed a qRT-PCR of Interferon beta and STAT1. The genes showed no differential expression in any of our transfections (Additional file 2).

### Effect of SGS on the apoptotic threshold in pancreatic cancer cells

To determine the optimal time point for harvesting and to show that all groups displayed a similar kinetic response to transfection, a dynamic measurement of caspases 3 and 7 activity was conducted every 12 hours after transfection; activation was maximal after 72 h (Additional file 1).

**Figure 2:**
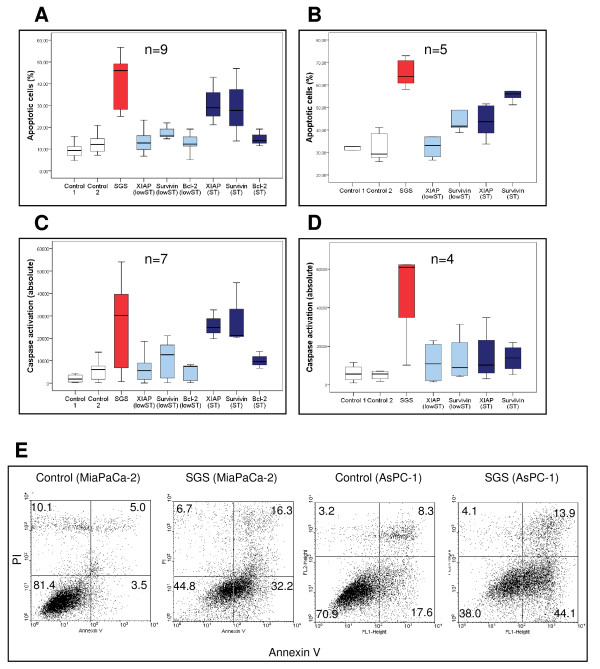
**Effect of SGS on apoptosis induction**. Apoptosis induction by SGS-transfected cells was measured in flow cytometry in MiaPaCa-2 cells (**A**) and AsPC-1 cells (**B) **(Control 1 = untreated cells; control 2 = cells treated with non-sense siRNA). Caspase activation 72 h after transfection in MiaPaCa-2 cells (**C**) and AsPC-1 cells (**D**) (Control 1 = untreated cells; control 2 = cells treated with non-sense siRNA). Examples of the FACS analysis of MiaPACa-2 (left) and AsPC-1 (right). Apoptotic cells are displayed in the upper and lower right quadrants (**E**).

Simultaneous silenced MiaPaCa-2 cells showed a 3.43-fold increase in the rate of apoptotic cells in flow cytometry compared to controls (12.81 ± 2.09 vs. 3.73 ± 0.55; *p *< 0.001). Standard single transfected cells (ST) of the Survivin group also resulted in a significantly elevated apoptosis rate compared to the controls (7.77 ± 1.82 vs. 3.73 ± 0.55; *p *= 0.043) (Figure 2A, E).

Accordingly, caspases 3 and 7 showed significant activity in the SGS group in MiaPaCa-2 cells compared to controls (9.95 ± 1.78 vs. 2.15 ± 0.27; *p *= 0.002). Single transfection of Survivin led to a significant caspase activation in the lowST (3.77 ± 0.56; p = 0.015) as well as in the ST group (8.4 ± 3.10; p = 0.007).

Moreover, transfection of XIAP led to significant caspase activation in the ST group (7.0 ± 0.35; p = 0.011) (Figure 2C) (Table 2).

**Table 2:** Effects of transfections on apoptosis induction

Target gene		MiaPaCa-2	AsPC-1
		mean (S.E.)	mean (S.E.)
Control	Annexin V	3.73 (± 0.5481)	2.23 (± 0.2232)
	Caspase	2.15 (± 0.2665)	1.17 (± 0.3060)
	Viable Cells	1.65 (± 0.2651)	1.25 (± 0.1784)

SGS	Annexin V	**12.81 **(± 2.0870)	**4.49 **(± 0.2966)
	Caspase	**9.96 **(± 1.7829)	**9.90 **(± 1.9189)
	Viable Cells	**6.70 **(± 2.2837)	**4.75 **(± 0.9908)

XIAP (low ST)	Annexin V	3.96 (± 0.6850)	2.34 (± 0.1797)
	Caspase	2.41 (± 0.5015)	2.24 (± 1.0513)
	Viable Cells	2.42 (± 1.0029)	1.20 (± 0.1452)

Survivin (low ST)	Annexin V	5.81 (± 1.0148)	**3.17 **(± 0.1986)
	Caspase	**3.77 **(± 0.5577)	2.77 (± 0.8111)
	Viable Cells	**4.02 **(± 0.8603)	**2.18 **(± 0.1375)

Bcl-2 (low ST)	Annexin V	3.40 (± 0.4986)	2.36 (± 0.1309)
	Caspase	1.88 (± 0.2001)	1.56 (± 0.4392)
	Viable Cells	2.27 (± 0.6989)	1.25 (± 0.1200)

XIAP (ST)	Annexin V	4.76 (± 0.7350)	**3.19 **(± 0.1122)
	Caspase	**7.00 **(± 0.3533)	2.55 (± 0.4072)
	Viable Cells	2.59 (± 0.7848)	1.87 (± 2.3098)

Survivin (ST)	Annexin V	**7.77 **(± 1.8167)	**3.94 **(± 0.2719)
	Caspase	**8.40 **(± 3.1037)	3.27 (± 0.9557)
	Viable Cells	**3.40 **(± 0.7485)	**5.23 **(± 2.3098)

Bcl-2 (ST)	Annexin V	3.51 (± 0.5311)	2.84 (± 0.1863)
	Caspase	2.94 (± 0.8894)	2.53 (± 1.1196)
	Viable Cells	1.49 (± 0.4988)	1.28 (± 0.1331)

Apoptosis induction was analyzed by Annexin V positivity, caspase activation and viable cell count. All results are normalized to non-treated cells. Viable cell counts are depicted in inverse ratios for better comparison. Significant results are marked in bold.

In AsPC-1 cells, SGS resulted in a 2.01-fold increase in the rate of apoptotic cells compared to controls as measured by flow cytometry (4.49 ± 0.30 vs. 2.23 ± 0.22; *p *= 0.009). Survivin showed increased numbers of apoptotic cells in flow cytometry in the lowST (3.17 ± 0.20; *p *= 0.0028) as well as in the ST group (3.94 ± 0.27; *p *= 0.009). ST of XIAP led to a significantly elevated apoptosis rate compared to controls (3.19 ± 0.11; *p *= 0.009) (Figure 2B, E).

Analogously, activation of caspases 3 and 7 in AsPC-1 cells was significantly elevated in the SGS group (9.90 ±1.92 vs. 1.17 ± 0.31; *p *= 0.021). However, none of the control groups showed statistically significant activation, although there was a tendency in the XIAP (2.55 ± 0.41) and Survivin ST groups (3.27 ± 0.96) (Figure 2D) (Table 2).

Corresponding to the higher apoptosis rate we saw a reduced number of viable cells in the SGS group in both cell lines, with a 6.7-fold reduction of vital MiaPaCa-2 cells (p = 0.021) and a 4.75-fold reduction in AsPC-1 cells (p = 0.05) as compared to controls (Table 2).

## Discussion

Apoptosis resistance in pancreatic cancer is highly relevant for the aggressive nature of the disease. Previous studies in pancreatic cancer have described multiple defects in apoptosis signaling at different levels of the pathway [8,23,24]. Based on the concept of apoptotic thresholds [9], we hypothesized that there might be a benefit in hitting multiple target genes at the same time rather than individual genes to achieve an effect on tumor survival. To test our hypothesis, we required candidate genes to be upregulated, synergistic, anti-apoptotic members of the cell death pathway.

The literature search identified different upregulated anti-apoptotic genes at the protein level in pancreatic carcinoma compared to normal tissue. XIAP, Survivin and Bcl-2 showed all required characteristics, like upregulation at the protein level and anti-apoptotic action [11,13]. Furthermore, all genes are members of the intrinsic pathway, which has special importance in pancreatic carcinoma [8,25].

SGS resulted in a statistically significant activation of apoptosis in tumor cells as determined by flow cytometry and caspase activation. None of the low-dose single transfections (lowST), which used the same dose of target siRNA, showed comparable effects on the phenotype of apoptosis resistance, although some effects were statistically significant.

In standard-dose single transfected (ST) cells, XIAP as well as Survivin showed apoptosis induction, but no group reached the effect achieved in the SGS group.

In AsPC-1 cells, the number of apoptotic cells in the ST group as measured by FACS analysis was not directly proportional to activation of caspases although there was a trend towards higher activation of caspases in the XIAP and Survivin ST group (Figure 2B and 2D). However, to our knowledge the relation between amount of activated caspases and extent of apoptosis induction is not necessarily linear and the trend towards higher activation in the Survivin group might be sufficient to result in the measured number of apoptotic cells. Furthermore, other effector caspases which were not measured by our assay might be involved in apoptosis induction in AsPc-1 cells.

**Figure 3:**
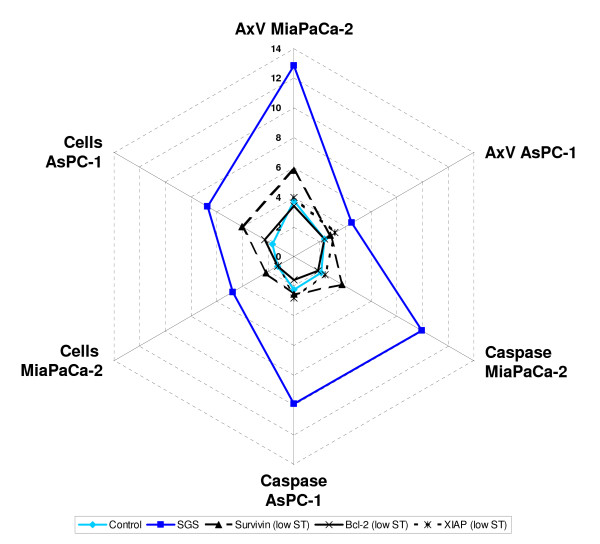
**Kiviat Diagram of SGS action in pancreatic cell lines**. SGS induced apoptosis in both cell lines measured either by Annexin V staining or by caspase activity. SGS also reduced the number of viable cells in both cell lines. For better visualization, we show the inverse ratio of the cell count. In each assay, SGS generated the highest signals (data were taken from table 2).

Although simultaneous silencing of the candidate genes was carried out with low dose transfection for each of the target genes we saw a significant effect on apoptosis induction. Contrary, the single transfections with lowST only resulted in subliminal effects on apoptosis induction (Figure 3).

This effect of SGS might be due to a reduction in the anti-apoptotic buffering capacity by summation of sub-threshold effects on the different, redundant control mechanisms. Our experiment therefore supports the model of an apoptotic threshold determined by the "net difference between its activity and opposing compensatory signals" [9]. This concept might be interesting for the design of future therapies. By choosing appropriate target genes it could be possible to selectively target pancreatic cancer cells while preserving normal tissue.

Another interesting aspect of our study is, that the single members do display a different order of precedence in their anti-apoptotic function. Although previous studies have shown important functions of all three genes in pancreatic cancer cells, the direct comparison shows that IAPs might have a more prominent position in apoptosis resistance in pancreatic cancer than Bcl-2.

## Conclusions

Pancreatic cancer shows a distinct apoptosis resistance which is due to numerous dysregulations within the cell death pathway. In the present study we performed a subliminal silencing of different anti-apoptotic target genes simultaneously, which had a significant impact on apoptosis induction in pancreatic cancer cells. Thus, we give further evidence for the concept of an anti-apoptotic buffering capacity of pancreatic cancer cells.

## Competing interests

The authors declare that they have no competing interests.

## Authors' contributions

FR Made substantial contributions to conception and design. He carried out transfection and western blotting and analysis and interpretation of data. NS carried out transfection, western blotting and qRT-PCR. She also was involved in FACS analysis and caspase assays. AKL carried out transfection, western blotting and qRT-PCR. She also was involved in FACS analysis and caspase assays. HDS participated in the design of the study and revised it critically for important intellectual content. RG participated in the design of the study and revised it critically for important intellectual content. CP conceived the study and participated in its design and coordination and helped to draft the manuscript. All authors have given final approval of the version to be published.

## Pre-publication history

The pre-publication history for this paper can be accessed here:

http://www.biomedcentral.com/1471-2407/10/379/prepub

## Supplementary Material

Additional file 1 **In AsPC-1 cells Bcl-2 expression was not detectable in different passages of the cells using western blot (A)**. We determined the optimal time point for harvesting by activation of caspase 3 and 7. The groups displayed a similar kinetic response to transfection. The dynamic measurement was conducted every 12 hours after transfection. All Caspase assays were done in duplicate (B). Click here for file

Additional file 2 **Expression of STAT1 (A) and Interferon-beta (B) in transfected cells**. AsPC1 cells are depicted in white, MiaPaCa-2 cells are in grey. All samples were normalized to β-Actin as house keeping gene. Click here for file
