# Supplementary material for: Simultaneous gene silencing of Bcl-2, XIAP and Survivin re-sensitizes pancreatic cancer cells towards apoptosis
Source: BMC Cancer. 2010 Jul 20;10:379. doi: 10.1186/1471-2407-10-379 (PMC2912871; doi:10.1186/1471-2407-10-379)
Supplement: Additional file 1 — In AsPC-1 cells Bcl-2 expression was not detectable in different passages of the cells using western blot (A). We determined the optimal time point for harvesting by activation of caspase 3 and 7. The groups displayed a similar kinetic response to transfection. The dynamic measurement was conducted every 12 hours after transfection. All Caspase assays were done in duplicate (B). [file 1471-2407-10-379-S1.PPT]

## Slide 1
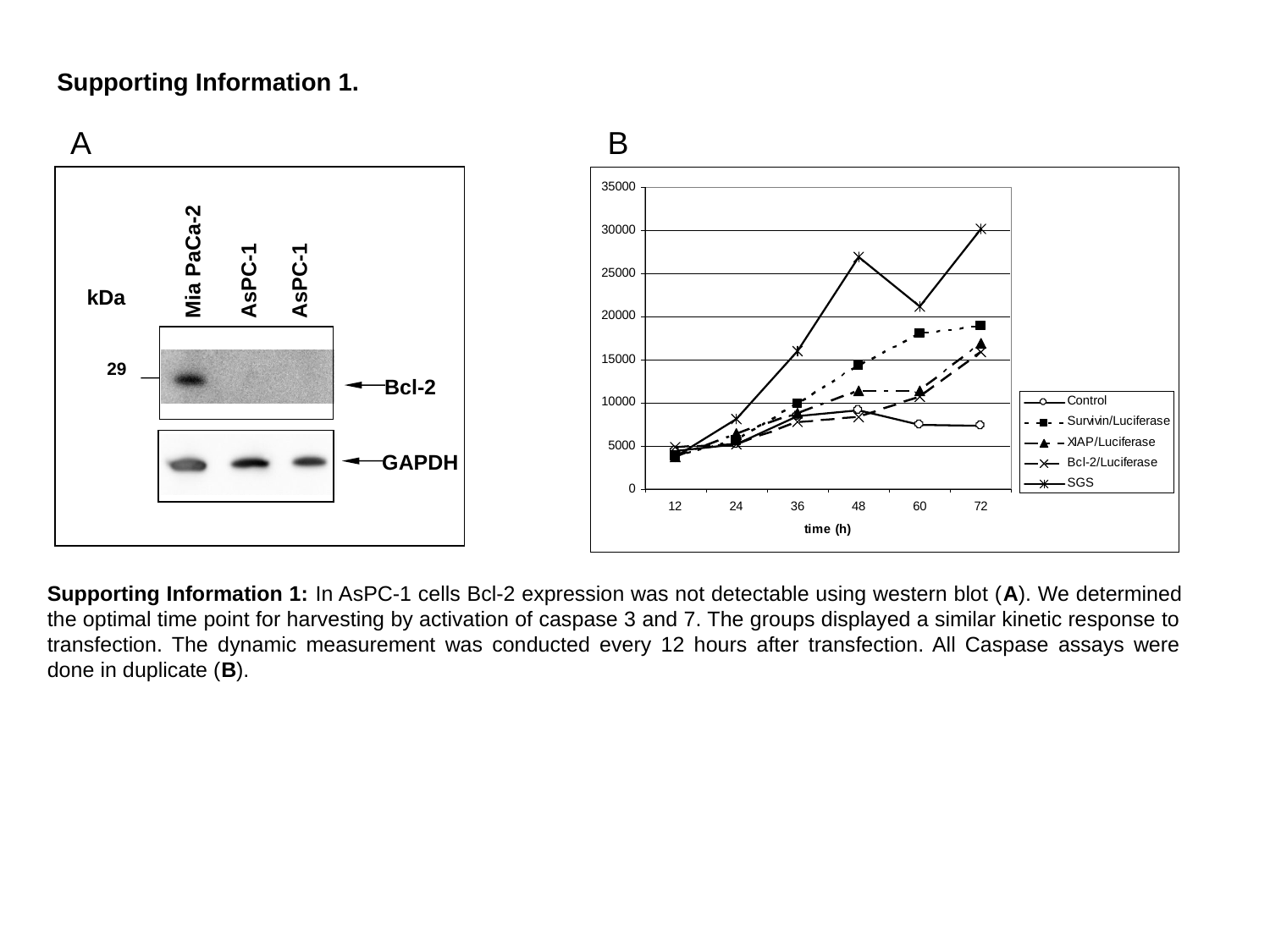

Supporting Information 1.
A
Mia PaCa-2
AsPC-1
AsPC-1
kDa
Bcl-2
29
GAPDH
B
Supporting Information 1: In AsPC-1 cells Bcl-2 expression was not detectable using western blot (A). We determined the optimal time point for harvesting by activation of caspase 3 and 7. The groups displayed a similar kinetic response to transfection. The dynamic measurement was conducted every 12 hours after transfection. All Caspase assays were done in duplicate (B).
