# Supplementary material for: Simultaneous gene silencing of Bcl-2, XIAP and Survivin re-sensitizes pancreatic cancer cells towards apoptosis
Source: BMC Cancer. 2010 Jul 20;10:379. doi: 10.1186/1471-2407-10-379 (PMC2912871; doi:10.1186/1471-2407-10-379)
Supplement: Additional file 2 — Expression of STAT1 (A) and Interferon-beta (B) in transfected cells. AsPC1 cells are depicted in white, MiaPaCa-2 cells are in grey. All samples were normalized to β-Actin as house keeping gene. [file 1471-2407-10-379-S2.PPT]

## Slide 1
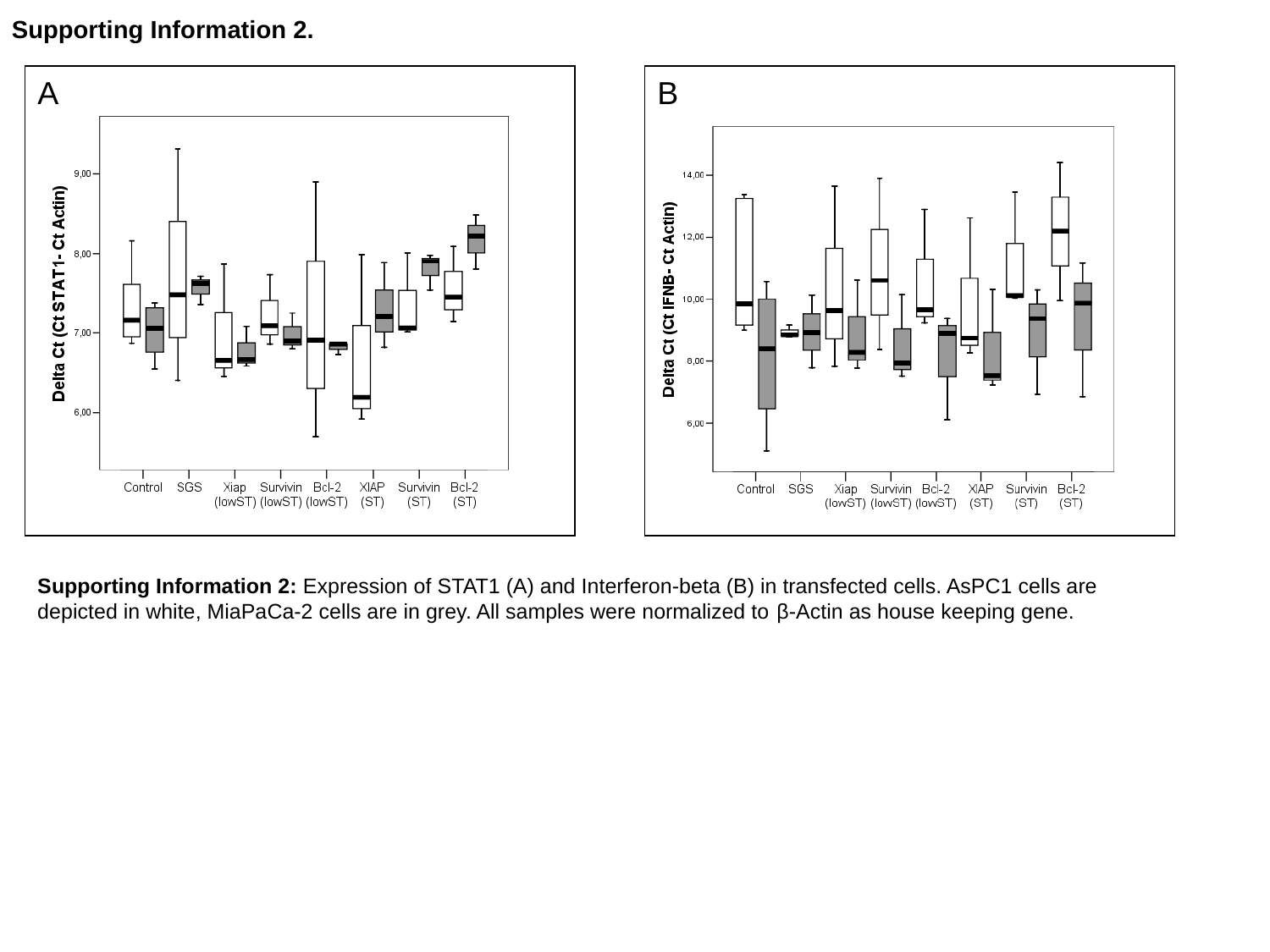

Supporting Information 2.
A
B
Supporting Information 2: Expression of STAT1 (A) and Interferon-beta (B) in transfected cells. AsPC1 cells are depicted in white, MiaPaCa-2 cells are in grey. All samples were normalized to β-Actin as house keeping gene.
